# (3*RS*)-*S*-[1-(3-Chloro­phen­yl)-2-oxopyr­roli­din-3-yl]thio­uronium bromide

**DOI:** 10.1107/S1600536809001603

**Published:** 2009-01-28

**Authors:** Jiří Hanusek, Miloš Sedlák, Pavel Drabina, Aleš Ružička

**Affiliations:** a Institute of Organic Chemistry and Technology, Faculty of Chemical Technology, University of Pardubice, nám. Čs. legií 565, Pardubice 532 10, Czech Republic; b Department of General and Inorganic Chemistry, Faculty of Chemical Technology, University of Pardubice, nám. Čs. legií 565, Pardubice 532 10, Czech Republic

## Abstract

In the title molecular salt, C_11_H_13_ClN_3_OS^+^·Br^−^, the C—N bond lengths in the –S–C(NH_2_)_2_ fragment indicate partial double-bond character of these bonds. The constituent ions are connected by N—H⋯Br bridges into Z-shaped chains. The supra­molecular architecture of the structure can be described by being composed of these chains inter­locked by additional C—H⋯Br short contacts. An intra­molecular N—H⋯O=C bridge, as well as weak C—H⋯O hydrogen bonds, are also present in the structure.

## Related literature

For the preparation and reactivity of isothiuronium salts, see: Hanusek *et al.* (2004 ▶); Sedlák *et al.* (2002 ▶, 2003 ▶). For related structures, see: Bel’skii *et al.* (1985 ▶); Cotton *et al.* (2006 ▶); Hanusek *et al.* (2009 ▶); Ishii *et al.* (2000 ▶); L’abbe *et al.* (1980 ▶); Luger *et al.* (1996 ▶); Rovnyak *et al.* (1995 ▶); Vijayan & Mani (1977 ▶).
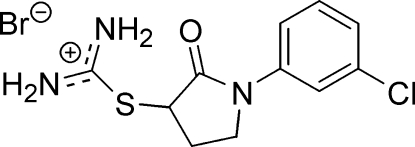


## Experimental

### 

#### Crystal data

C_11_H_13_ClN_3_OS^+^·Br^−^
*M* *_r_* = 350.66
Monoclinic, 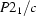

*a* = 15.7379 (9) Å
*b* = 6.4250 (5) Å
*c* = 15.0531 (7) Å
β = 117.329 (5)°
*V* = 1352.22 (16) Å^3^
*Z* = 4
Mo *K*α radiation
μ = 3.38 mm^−1^
*T* = 150 (2) K
0.29 × 0.12 × 0.03 mm

#### Data collection

Bruker–Nonius KappaCCD diffractometer
Absorption correction: gaussian (Coppens, 1970 ▶) *T*_min_ = 0.542, *T*_max_ = 0.873
15769 measured reflections
3095 independent reflections
2214 reflections with *I* > 2σ(*I*)
*R*_int_ = 0.097

#### Refinement

*R*[*F* ^2^ > 2σ(*F* ^2^)] = 0.050
*wR*(*F* ^2^) = 0.096
*S* = 1.15
3095 reflections
164 parameters
H-atom parameters constrained
Δρ_max_ = 0.48 e Å^−3^
Δρ_min_ = −0.47 e Å^−3^

### 

Data collection: *COLLECT* (Hooft, 1998 ▶) and *DENZO* (Otwin­owski & Minor, 1997 ▶); cell refinement: *COLLECT* and *DENZO*; data reduction: *COLLECT* and *DENZO*; program(s) used to solve structure: *SIR92* (Altomare *et al.*, 1994 ▶); program(s) used to refine structure: *SHELXL97* (Sheldrick, 2008 ▶); molecular graphics: *PLATON* (Spek, 2003 ▶); software used to prepare material for publication: *SHELXL97*.

## Supplementary Material

Crystal structure: contains datablocks I, global. DOI: 10.1107/S1600536809001603/fb2122sup1.cif

Structure factors: contains datablocks I. DOI: 10.1107/S1600536809001603/fb2122Isup2.hkl

Additional supplementary materials:  crystallographic information; 3D view; checkCIF report

## Figures and Tables

**Table 1:** Hydrogen-bond geometry (Å, °)

*D*—H⋯*A*	*D*—H	H⋯*A*	*D*⋯*A*	*D*—H⋯*A*
N2*A*—H2*AB*⋯Br1	0.88	2.43	3.290 (3)	166
N2*A*—H2*AA*⋯Br1^i^	0.88	2.68	3.452 (3)	147
N2*A*—H2*AA*⋯Br1^ii^	0.88	2.91	3.426 (3)	119
N3*A*—H3*AA*⋯Br1^i^	0.88	2.44	3.273 (3)	158
N3*A*—H3*AB*⋯O1	0.88	2.12	2.823 (4)	137
C2—H2*A*⋯O1^iii^	0.99	2.57	3.352 (5)	137
C3—H3*B*⋯Br1^iv^	0.99	3.01	3.737 (4)	131
C10—H10⋯O1	0.95	2.22	2.852 (5)	124

Symmetry codes: (i) 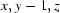; (ii) 
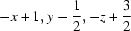
; (iii) 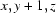; (iv) 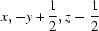.

**Fig. 1.**
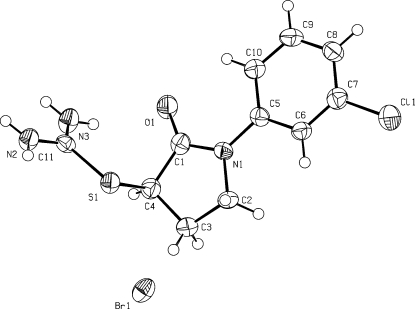
View of the title molecule with the displacement ellipsoids shown on 50% probability level. The H atoms are shown with arbitrary radius.

**Fig. 2.**
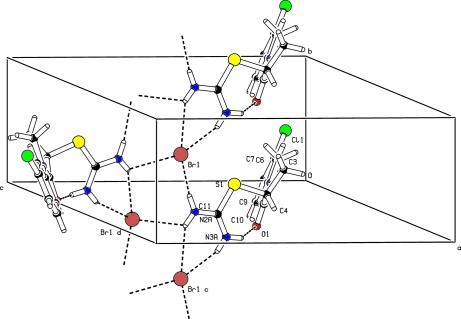
View of the motif of the structure with the hydrogen bonding.

**Fig. 3.**
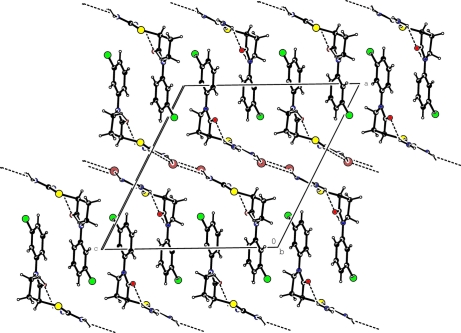
View of the unit cell along the axis c. The H bonding is depicted. Symmetry codes for Br1c: x, -1+y, z; for Br1d: 1-x, -1/2+y, 3/2-z; for another isothiuronium fragment: x, 1+y, z.

## supplementary crystallographic information

### Comment

In our previous papers (Sedlák *et al.*, 2002, 2003; Hanusek *et al.*, 2004) we have reported that substituted *S*-(1-phenylpyrrolidin-2-on-3-yl)isothiuronium salts undergo an intramolecular recyclization reaction in a weak basic medium. During this reaction the γ-lactam cycle is split while a thiazolidine cycle, *i.e.* a substituted 2-imino-5-[2-(phenylamino)ethyl]thiazolidin-4-one, is formed.

A further interest has been provoked by a discovery (Sedlák *et al.*, 2002, 2003; Hanusek *et al.*, 2004) that *N*-unsubstituted isothiuronium salts undergo a transformation involving only a general base catalysis whereas in the case of *N*,*N*'-dimethylisothiuronium salts such a transformation is catalyzed by both acid and base buffer components.

In continuation of our above mentioned studies, the crystal structures of (3*RS*)-*S*-[1-(3-chlorophenyl)pyrrolidin-2-on-3-yl]isothiuronium bromide (Scheme 1, Figs. 1-3) and its *N*,*N*'-dimethyl derivative (Hanusek *et al.*, 2009) were determined and the influence of the methyl substituents bound to the isothiuronium moiety was examined.

There is a limited number of X-ray structures of isothiouronium salts reported in the literature. Eleven of these are the compounds of the S–C(NH_2_)_2_ type with no replacement of the hydrogens that are pertinent to the –NH_2_ group (*e.g.* Vijayan *et al.*, 1977; Bel'skii *et al.*, 1985; Ishii *et al.*, 2000). The rest are the compounds where both nitrogen atoms are connected to a chain thus forming five- to eight-membered rings (*e.g.* Rovnyak *et al.*, 1995; Cotton *et al.*, 2006; Luger *et al.*, 1996). There is only one example of a disubstituted acyclic species, *N*-methyl- *N*'-phenyl-*S*-sulfomethylisothiourea (L'abbe *et al.*, 1980).

In the title structure and its *N*,*N*'-dimethyl derivative the respective interplanar angles between the S–C(NHR)_2_ group and the heterocyclic rings are almost the same: 66.7 (1) ° and 71.3 (1) °. The S1–C11 distance (1.749 (4) Å) in the title structure fits to the literature range 1.717–1.760 Å - *cf.* 1.770 (4) Å in *N*,*N*'-dimethyl derivative. The C11–N2 and C11–N3 distances also agree to the literature range that is 1.258 - 1.326 Å. (In the title compound these distances are 1.312 (5), 1.296 (5) Å, respectively, while  in the *N*,*N*'-dimethyl derivative these distances are 1.308 (5), 1.308 (5) Å, respectively).

These C-N bond lengths in the S–C(NH_2_)_2_ fragment of the title structure reveal a partly double bond character of these bonds. This is in accordance with planarity of this fragment. (The groups -NH_2_ were therefore duly constrained during refinement.)

The interatomic angles C4–S1–C11 (104.56 (18)°) and N2–C11–N3 (122.0 (4)°) in the title compound are similar to those in the related *N*,*N*'-dimethyl derivative (98.75 (18)° and 122.6 (4)°, respectively) and also fall into the literature range which is 99.2–105.5° and 108.0—123.5°, respectively. The twist angles about the N1–C5 bonds, which show a mutual orientation of both rings, are  7.8 (1) and 29.7 (1) ° in the title compound and its *N*,*N*'-dimethyl derivative, respectively.

All the known isothiouronium cations reveal hydrogen bonding in the crystal structure. Also in the packing of the molecules of the title compound and its *N*,*N*'-dimethyl derivative such interactions are present. All the NH_2_ hydrogen atoms are donated to the bromine atoms except for H3AB. Those with the shortest Br···H distances form infinite chains *via* the N2-H2AB···Br···H3AA-N3 motifs (Figs. 2 and 3; Tab. 1) with the angle H2AB···Br1···H3AA equal to 100.7 (1)°. There is also present an intramolecular  N–H···O=C contact (Tab. 1, Fig. 2). The crystal packing can be described as H-bonded interlocked *Z*- shaped ribbons caused by the presence of additional short contacts C–H···Br (Fig. 3).

### Experimental

The title compound was synthesized according to Hanusek *et al.* (2004) from saturated acetone solutions of the racemic 3-bromo-1-(3-chlorophenyl)pyrrolidin-2-one and thiourea. Suitable single crystals (plates) were grown directly from the reaction mixture. Their average size was 0.2×0.1×0.02 mm.

### Refinement

All the hydrogens were discernible in the difference electron density map. However, all the hydrogens were situated into idealized positions and refined riding on their parent C or N atoms, with N–H = 0.88 Å, U(H) = 1.2U*_eq_*(C), C–H = 0.95Å (for aryl H), C–H = 0.99 Å for methylene and C–H = 1.00 Å for methine, U(H) = 1.2U*_eq_*(C/N) for the amine, methylene and methine H atoms, respectively.

### Crystal data

**Table:**

C_11_H_13_ClN_3_OS^+^·Br^−^	*F*(000) = 704
*M**_r_* = 350.66	*D*_x_ = 1.722 Mg m^−^^3^
Monoclinic, *P*2_1_/*c*	Mo *K*α radiation, λ = 0.71073 Å
Hall symbol: -P 2ybc	Cell parameters from 15779 reflections
*a* = 15.7379 (9) Å	θ = 1–27.5°
*b* = 6.4250 (5) Å	µ = 3.38 mm^−^^1^
*c* = 15.0531 (7) Å	*T* = 150 K
β = 117.329 (5)°	Plate, colourless
*V* = 1352.22 (16) Å^3^	0.29 × 0.12 × 0.03 mm
*Z* = 4	

### Data collection

**Table:**

Bruker–Nonius KappaCCD diffractometer	3095 independent reflections
Radiation source: fine-focus sealed tube	2214 reflections with *I* > 2σ(*I*)
graphite	*R*_int_ = 0.097
Detector resolution: 9.091 pixels mm^-1^	θ_max_ = 27.5°, θ_min_ = 2.9°
φ and ω scans	*h* = −20→20
Absorption correction: gaussian (Coppens, 1970)	*k* = −7→8
*T*_min_ = 0.542, *T*_max_ = 0.873	*l* = −19→19
15769 measured reflections	

### Refinement

**Table:**

Refinement on *F*^2^	Secondary atom site location: difference Fourier map
Least-squares matrix: full	Hydrogen site location: difference Fourier map
*R*[*F*^2^ > 2σ(*F*^2^)] = 0.050	H-atom parameters constrained
*wR*(*F*^2^) = 0.096	*w* = 1/[σ^2^(*F*_o_^2^) + (0.0236*P*)^2^ + 1.6718*P*] where *P* = (*F*_o_^2^ + 2*F*_c_^2^)/3
*S* = 1.15	(Δ/σ)_max_ < 0.001
3095 reflections	Δρ_max_ = 0.48 e Å^−^^3^
164 parameters	Δρ_min_ = −0.47 e Å^−^^3^
0 restraints	Extinction correction: *SHELXL97* (Sheldrick, 2008), Fc^*^=kFc[1+0.001xFc^2^λ^3^/sin(2θ)]^-1/4^
52 constraints	Extinction coefficient: 0.0036 (5)
Primary atom site location: structure-invariant direct methods	

### Special details

**Table:**

Geometry. All esds (except the esd in the dihedral angle between two l.s. planes) are estimated using the full covariance matrix. The cell esds are taken into account individually in the estimation of esds in distances, angles and torsion angles; correlations between esds in cell parameters are only used when they are defined by crystal symmetry. An approximate (isotropic) treatment of cell esds is used for estimating esds involving l.s. planes.
Refinement. Refinement of F^2^ against ALL reflections. The weighted R-factor wR and goodness of fit S are based on F^2^, conventional R-factors R are based on F, with F set to zero for negative F^2^. The threshold expression of F^2^ > 2sigma(F^2^) is used only for calculating R-factors(gt) etc. and is not relevant to the choice of reflections for refinement. R-factors based on F^2^ are statistically about twice as large as those based on F, and R- factors based on ALL data will be even larger.

### Fractional atomic coordinates and isotropic or equivalent isotropic displacement parameters (Å^2^)

**Table:**

	*x*	*y*	*z*	*U*_iso_*/*U*_eq_	
Br1	0.48049 (3)	0.45589 (6)	0.65892 (3)	0.04086 (16)	
S1	0.34553 (7)	0.14593 (15)	0.41198 (8)	0.0339 (2)	
Cl1	−0.19436 (7)	0.30572 (19)	−0.02926 (9)	0.0532 (3)	
O1	0.21265 (19)	−0.2562 (4)	0.2711 (2)	0.0450 (7)	
C5	0.0464 (3)	0.0076 (6)	0.1482 (3)	0.0295 (8)	
C6	−0.0184 (3)	0.1577 (6)	0.0890 (3)	0.0330 (9)	
H6	0.0040	0.2871	0.0774	0.040*	
C4	0.3119 (3)	0.0455 (6)	0.2870 (3)	0.0346 (9)	
H4	0.3640	−0.0404	0.2852	0.042*	
C11	0.3880 (2)	−0.0716 (6)	0.4901 (3)	0.0291 (8)	
C2	0.1792 (3)	0.2598 (6)	0.1780 (3)	0.0372 (10)	
H2A	0.1660	0.3657	0.2179	0.045*	
H2B	0.1480	0.3027	0.1069	0.045*	
C1	0.2191 (3)	−0.0747 (6)	0.2493 (3)	0.0329 (9)	
C10	0.0120 (3)	−0.1790 (7)	0.1662 (3)	0.0398 (10)	
H10	0.0552	−0.2828	0.2072	0.048*	
C8	−0.1500 (3)	−0.0645 (7)	0.0639 (3)	0.0422 (10)	
H8	−0.2169	−0.0891	0.0349	0.051*	
C7	−0.1148 (3)	0.1186 (7)	0.0473 (3)	0.0375 (9)	
C9	−0.0854 (3)	−0.2119 (7)	0.1239 (3)	0.0459 (11)	
H9	−0.1085	−0.3394	0.1364	0.055*	
C3	0.2862 (3)	0.2309 (7)	0.2163 (3)	0.0410 (10)	
H3A	0.3216	0.3568	0.2522	0.049*	
H3B	0.3012	0.2018	0.1605	0.049*	
N2A	0.4287 (2)	−0.0308 (5)	0.5862 (2)	0.0359 (8)	
H2AA	0.4517	−0.1329	0.6299	0.043*	
H2AB	0.4331	0.0986	0.6070	0.043*	
N1	0.1455 (2)	0.0527 (5)	0.1902 (2)	0.0301 (7)	
N3A	0.3800 (2)	−0.2598 (5)	0.4563 (3)	0.0387 (8)	
H3AA	0.4023	−0.3650	0.4982	0.046*	
H3AB	0.3523	−0.2825	0.3914	0.046*	

### Atomic displacement parameters (Å^2^)

**Table:**

	*U*^11^	*U*^22^	*U*^33^	*U*^12^	*U*^13^	*U*^23^
Br1	0.0583 (3)	0.0267 (2)	0.0360 (2)	−0.00529 (19)	0.0203 (2)	−0.00053 (19)
S1	0.0371 (5)	0.0258 (5)	0.0328 (5)	0.0008 (4)	0.0109 (4)	−0.0016 (4)
Cl1	0.0390 (6)	0.0562 (7)	0.0584 (7)	0.0075 (5)	0.0172 (5)	0.0116 (6)
O1	0.0468 (17)	0.0269 (16)	0.0439 (18)	−0.0033 (13)	0.0059 (13)	0.0010 (13)
C5	0.032 (2)	0.030 (2)	0.027 (2)	−0.0057 (15)	0.0133 (16)	−0.0075 (16)
C6	0.036 (2)	0.030 (2)	0.034 (2)	−0.0026 (17)	0.0161 (17)	0.0017 (17)
C4	0.034 (2)	0.037 (2)	0.032 (2)	−0.0007 (18)	0.0144 (17)	0.0001 (19)
C11	0.0234 (18)	0.025 (2)	0.039 (2)	−0.0001 (15)	0.0141 (16)	−0.0007 (17)
C2	0.037 (2)	0.030 (2)	0.040 (2)	−0.0071 (17)	0.0141 (18)	0.0067 (18)
C1	0.038 (2)	0.030 (2)	0.027 (2)	0.0000 (17)	0.0123 (17)	−0.0043 (17)
C10	0.040 (2)	0.036 (2)	0.038 (2)	−0.0058 (18)	0.0143 (19)	0.0043 (19)
C8	0.035 (2)	0.056 (3)	0.034 (2)	−0.014 (2)	0.0140 (18)	−0.002 (2)
C7	0.038 (2)	0.043 (2)	0.033 (2)	0.0007 (19)	0.0176 (18)	−0.0003 (19)
C9	0.048 (2)	0.041 (3)	0.047 (3)	−0.015 (2)	0.020 (2)	0.004 (2)
C3	0.038 (2)	0.048 (3)	0.038 (2)	−0.0066 (19)	0.0177 (19)	0.007 (2)
N2A	0.0434 (19)	0.0261 (16)	0.0307 (19)	−0.0003 (15)	0.0104 (15)	−0.0017 (15)
N1	0.0291 (16)	0.0265 (16)	0.0319 (18)	−0.0045 (14)	0.0116 (14)	−0.0010 (14)
N3A	0.049 (2)	0.0262 (18)	0.0327 (19)	0.0049 (15)	0.0117 (16)	−0.0026 (15)

### Geometric parameters (Å, °)

**Table:**

S1—C11	1.749 (4)	C2—H2A	0.9900
S1—C4	1.822 (4)	C2—H2B	0.9900
Cl1—C7	1.737 (4)	C1—N1	1.363 (5)
O1—C1	1.228 (4)	C10—C9	1.379 (6)
C5—C6	1.389 (5)	C10—H10	0.9500
C5—C10	1.392 (5)	C8—C7	1.371 (6)
C5—N1	1.418 (4)	C8—C9	1.380 (6)
C6—C7	1.374 (5)	C8—H8	0.9500
C6—H6	0.9500	C9—H9	0.9500
C4—C1	1.514 (5)	C3—H3A	0.9900
C4—C3	1.523 (5)	C3—H3B	0.9900
C4—H4	1.0000	N2A—H2AA	0.8800
C11—N3A	1.296 (5)	N2A—H2AB	0.8800
C11—N2A	1.312 (5)	N3A—H3AA	0.8800
C2—N1	1.475 (5)	N3A—H3AB	0.8800
C2—C3	1.520 (5)		
			
C11—S1—C4	104.56 (18)	C9—C10—H10	120.3
C6—C5—C10	119.1 (3)	C5—C10—H10	120.3
C6—C5—N1	118.5 (3)	C7—C8—C9	118.0 (4)
C10—C5—N1	122.4 (3)	C7—C8—H8	121.0
C7—C6—C5	119.9 (4)	C9—C8—H8	121.0
C7—C6—H6	120.1	C8—C7—C6	121.9 (4)
C5—C6—H6	120.1	C8—C7—Cl1	119.1 (3)
C1—C4—C3	103.7 (3)	C6—C7—Cl1	119.0 (3)
C1—C4—S1	109.9 (3)	C10—C9—C8	121.8 (4)
C3—C4—S1	107.6 (3)	C10—C9—H9	119.1
C1—C4—H4	111.8	C8—C9—H9	119.1
C3—C4—H4	111.8	C2—C3—C4	104.7 (3)
S1—C4—H4	111.8	C2—C3—H3A	110.8
N3A—C11—N2A	122.0 (4)	C4—C3—H3A	110.8
N3A—C11—S1	122.9 (3)	C2—C3—H3B	110.8
N2A—C11—S1	115.1 (3)	C4—C3—H3B	110.8
N1—C2—C3	104.0 (3)	H3A—C3—H3B	108.9
N1—C2—H2A	111.0	C11—N2A—H2AA	120.0
C3—C2—H2A	111.0	C11—N2A—H2AB	120.0
N1—C2—H2B	111.0	H2AA—N2A—H2AB	120.0
C3—C2—H2B	111.0	C1—N1—C5	126.8 (3)
H2A—C2—H2B	109.0	C1—N1—C2	112.1 (3)
O1—C1—N1	126.5 (4)	C5—N1—C2	120.9 (3)
O1—C1—C4	124.6 (4)	C11—N3A—H3AA	120.0
N1—C1—C4	108.8 (3)	C11—N3A—H3AB	120.0
C9—C10—C5	119.4 (4)	H3AA—N3A—H3AB	120.0

### Hydrogen-bond geometry (Å, °)

**Table:**

*D*—H···*A*	*D*—H	H···*A*	*D*···*A*	*D*—H···*A*
N2A—H2AB···Br1	0.88	2.43	3.290 (3)	166
N2A—H2AA···Br1^i^	0.88	2.68	3.452 (3)	147
N2A—H2AA···Br1^ii^	0.88	2.91	3.426 (3)	119
N3A—H3AA···Br1^i^	0.88	2.44	3.273 (3)	158
N3A—H3AB···O1	0.88	2.12	2.823 (4)	137
C2—H2A···O1^iii^	0.99	2.57	3.352 (5)	137
C3—H3B···Br1^iv^	0.99	3.01	3.737 (4)	131
C10—H10···O1	0.95	2.22	2.852 (5)	124

Symmetry codes: (i) *x*, *y*−1, *z*; (ii) −*x*+1, *y*−1/2, −*z*+3/2; (iii) *x*, *y*+1, *z*; (iv) *x*, −*y*+1/2, *z*−1/2.

## References

[bb1] Altomare, A., Cascarano, G., Giacovazzo, C., Guagliardi, A., Burla, M. C., Polidori, G. & Camalli, M. (1994). *J. Appl. Cryst.* **27**, 435.

[bb2] Bel’skii, V. K., Babilev, F. V., Tryapitsyna, T. P. & Mukhin, E. A. (1985). *Dokl. Akad. Nauk SSSR*, **282**, 605–607.

[bb3] Coppens, P. (1970). *Crystallographic Computing*, edited by F. R. Ahmed, S. R. Hall & C. P. Huber, pp. 255–270. Copenhagen: Munksgaard.

[bb4] Cotton, F. A., Murillo, C. A., Wang, X. & Wilkinson, C. C. (2006). *Dalton Trans.* pp. 4623–4631. 10.1039/b608422b 17016574

[bb5] Hanusek, J., Hejtmánková, L., Štěrba, V. & Sedlák, M. (2004). *Org. Biomol. Chem.* **2**, 1756–1763. 10.1039/b401866d 15188043

[bb6] Hanusek, J., Sedlák, M., Drabina, P. & Ružička, A. (2009). *Acta Cryst.* E**65**, o413. 10.1107/S1600536809002153 PMC2968153 21582004

[bb7] Hooft, R. W. (1998). *COLLECT* Nonius, Delft, The Netherlands.

[bb8] Ishii, Y., Matsunaka, K. & Sakaguchi, S. (2000). *J. Am. Chem. Soc.* **122**, 7390–7391.

[bb9] L’abbe, G., Willocx, A., Toppet, S., Declercq, J. P., Germain, G. & van Meerssche, M. (1980). *Bull. Soc. Chim. Belg.* **89**, 487–488.

[bb10] Luger, P., Daneck, K., Engel, W., Trummlitz, G. & Wagner, K. (1996). *Eur. J. Pharm. Sci.* **4**, 175–187.

[bb11] Otwinowski, Z. & Minor, W. (1997). *Methods in Enzymology*, Vol. 276, *Macromolecular Crystallography*, Part A, edited by C. W. Carter Jr & R. M. Sweet, pp. 307–326. New York: Academic Press.

[bb12] Rovnyak, G. C., Kimball, S. D., Beyer, B., Cucinotta, G., Di Marco, J. D., Gougoutas, J., Hedberg, A., Malley, M., McCarthy, J. P., Zhang, R. & Moreland, S. (1995). *J. Med. Chem.* **38**, 119–129. 10.1021/jm00001a017 7837222

[bb13] Sedlák, M., Hanusek, J., Hejtmánková, L. & Kašparová, P. (2003). *Org. Biomol. Chem.* **1**, 1204–1209. 10.1039/b209107k 12926396

[bb14] Sedlák, M., Hejtmánková, L., Hanusek, J. & Macháček, V. (2002). *J. Heterocycl. Chem.* **39**, 1105–1107.

[bb15] Sheldrick, G. M. (2008). *Acta Cryst.* A**64**, 112–122. 10.1107/S0108767307043930 18156677

[bb16] Spek, A. L. (2003). *J. Appl. Cryst.* **36**, 7–13.

[bb17] Vijayan, K. & Mani, A. (1977). *Acta Cryst.* B**33**, 279–280.

